# Early Nutritional Patterns and Metabolic Biomarkers Associated with ROP Severity

**DOI:** 10.3390/medicina62010095

**Published:** 2026-01-01

**Authors:** Laura Bujoreanu Bezman, Carmen Tiutiuca, Florin Ciprian Bujoreanu, Mariana Stuparu-Crețu, Mădălina Nicoleta Matei, Dana Tutunaru, Alina Mihaela Călin, Florentin Dimofte, Elena Niculeț, Aurel Nechita

**Affiliations:** 1 Department of Ophthalmology, “Sf. Ioan” Emergency Clinical Hospital for Children, 800487 Galati, Romania; laura.bezman@ugal.ro; 2 Faculty of Medicine and Pharmacy, “Dunarea de Jos” University of Galati, 800385 Galati, Romania; mariana.stuparu@ugal.ro (M.S.-C.); madalina.matei@ugal.ro (M.N.M.); dana.tutunaru@ugal.ro (D.T.); alina.calin@ugal.ro (A.M.C.); florentin.dimofte@ugal.ro (F.D.); elena.niculet@ugal.ro (E.N.); aurel.nechita@ugal.ro (A.N.); 3 Department of Ophthalmology, “Sf. Apostol Andrei” Emergency Clinical Hospital, 800578 Galati, Romania

**Keywords:** retinopathy of prematurity (ROP), metabolic biomarkers, nutritional patterns, ROP severity, glycemic instability

## Abstract

*Background and Objectives:* Retinopathy of prematurity (ROP) remains a leading cause of preventable childhood blindness, with its severity influenced by a complex interaction between nutritional status, metabolic maturation, and systemic vulnerability. This study aimed to evaluate whether early nutritional patterns and serum metabolic parameters, including hepatic and renal biomarkers, are associated with ROP severity and whether they may serve as potential predictors of disease progression. *Materials and Methods:* We conducted a retrospective study on 140 preterm infants, totaling 280 eyes, admitted between 2021 and 2024 in two neonatal intensive care units (NICU). Each eye was analyzed independently according to International Classification of Retinopathy of Prematurity (ICROP) criteria. Data on the timing of enteral feeding, duration and type of nutrition, and serum levels of alanine aminotransferase (ALT), aspartate aminotransferase (AST), total protein, blood glucose, urea and creatinine were collected throughout the first 28 days of life. Statistical analysis included Kruskal–Wallis and Chi-square tests, with a significance threshold of *p* < 0.05. *Results:* ROP was identified in 53.57% of cases. Enteral feeding began earlier in infants without ROP, whereas delayed initiation and prolonged parenteral nutrition were associated with more advanced stages. Natural feeding decreased with increasing severity and was absent in aggressive retinopathy of prematurity (A-ROP). Severe disease stages showed higher AST, ALT, urea and creatinine levels, along with lower early total protein values. Glycemic instability was observed more frequently in stage 2 and stage 3. *Conclusions:* Early nutritional support, especially early enteral feeding and natural feeding, appears protective against ROP progression. Hepatic, renal and glycemic metabolic changes are closely correlated with disease severity, indicating that metabolic balance reflects overall vulnerability in preterm infants. Incorporating nutritional and metabolic assessment into routine screening may enhance early risk identification and optimize clinical monitoring.

## 1. Introduction

Retinopathy of prematurity (ROP) is one of the most frequent complications of prematurity, characterized by the abnormal development of retinal blood vessels. This retinal vulnerability is determined by the fact that the vessels are incompletely developed at birth, since the normal process of retinal vascularization is completed only around 40 weeks of gestational age (GA) [1,2]. Retinal neovascularization represents the central pathophysiological mechanism of the disease, being favored by vascular immaturity and by factors such as low GA, low birth weight (LBW), and oxygen administration [3].

In recent years, the incidence of severe forms has shown a significant increase, as advances in neonatal intensive care have led to greater survival among extremely low birth weight (ELBW) preterm infants. In these cases, in the absence of timely treatment, patients face a major risk of permanent visual deficits, which may progress to retinal detachment and even blindness [4,5]. According to the literature, ROP remains one of the worldwide leading causes of childhood blindness, being, after cortical visual impairment, the second most common cause of pediatric blindness [6,7].

Early detection through screening programs, together with the prompt initiation of treatment when indicated, represents an essential goal in preventing these severe complications [8]. At the same time, in addition to the increasing number of severe forms, the limited resources available for screening represent a major challenge for healthcare systems [9]. More than 80 years after its first description, ROP continues to be a central topic of research, as its risk factors are still not fully understood [10]. Understanding this pathology requires the analysis of pathophysiological mechanisms and the complex interaction between growth factors and metabolic balance, elements that directly influence retinal angiogenesis. Such a perspective is essential not only for limiting severe complications but also for improving the prediction of ROP risk, which would allow for more efficient screening programs focused on high-risk cases and, consequently, the optimization of medical resource utilization [11,12].

Vascular Endothelial Growth Factor (VEGF) is the key factor in retinal angiogenesis, but its action depends on the circulating levels of Insulin-like Growth Factor 1 (IGF-1) [13]. The role of IGF-1 is essential in the pathogenesis of ROP and manifests differently in the two phases of the disease. In preterm infants, during the first phase, immediately after birth, serum levels of IGF-1 decrease significantly due to the interruption of transplacental supply and the reduced capacity for endogenous synthesis. The deficit of IGF-1 disrupts the normal development of retinal vascularization and leads to the appearance of extensive avascular areas. In the second phase, as the avascular retina becomes hypoxic, VEGF secretion increases substantially. However, VEGF-driven angiogenesis is strongly modulated by IGF-1 availability. As IGF-1 levels rise with the infant’s postnatal maturation, IGF-1 can permissively support VEGF signaling and endothelial cell survival, thereby facilitating pathologic neovascularization in a VEGF-rich, hypoxic retinal environment. Therefore, IGF-1 has a dual and apparently paradoxical role: the early deficit blocks physiological angiogenesis, whereas later recovery in the setting of hypoxia may promote abnormal vessel growth [14,15].

This interaction between IGF-1 and VEGF shows that the development of retinal vascularization is not an isolated process. It depends directly on metabolic balance and nutritional intake [16,17]. In this context, the liver plays a central role, being the main endogenous source of IGF-1, while its function is influenced by caloric and protein intake as well as by the metabolic maturation of the preterm infant [18]. Hepatic metabolic activity is reflected by serum transaminase levels, enzymes involved in amino acid metabolism and cellular energy processes. In preterm infants, variations in these parameters may indicate the degree of hepatic maturity and the body’s ability to sustain protein synthesis and regulate growth factors, including IGF-1 [19,20]. It has also been demonstrated that breast milk contains IGF-1, suggesting that natural feeding may further support growth and angiogenic processes [21].

Glucose represents the main source of energy for the developing retina, especially for photoreceptors, which predominantly metabolize it through aerobic glycolysis despite their high mitochondrial density [22]. Glucose delivery to retinal tissues is tightly regulated by the blood–retinal barrier. The inner retina is supplied by the retinal circulation, whereas the outer retina is supplied by the choroidal circulation via the retinal pigment epithelium, with GLUT1 acting as a key transporter at both the inner and outer barriers [23]. In the context of prematurity, metabolic regulatory mechanisms are immature, and fluctuations in glucose levels are frequent [24]. Both hypoglycemia and hyperglycemia can interfere with retinal vascular development, hypoglycemia by limiting the available energy substrate and hyperglycemia through oxidative stress, inflammation, and altered expression of growth factors such as IGF-1 and VEGF [25,26]. Maintaining glycemic homeostasis during the first weeks of life is therefore essential for balanced retinal angiogenesis and for limiting the risk of pathological neovascularization [27].

The metabolic status of the preterm infant is also influenced by renal function, reflected by serum urea and creatinine levels [28]. The development of the RPE and the formation of the glomerular filtration membrane occur within a similar embryologic window, around the 4th–5th weeks of gestation [29,30]. Both structures depend on adequate vascular perfusion, and their early maturation makes them highly sensitive to perturbations in the extrauterine environment. Urea is closely related to protein metabolism and nitrogen balance, depending on both enteral and parenteral nutrition [31]. Creatinine, in turn, reflects the degree of renal maturity during the first weeks of life and the intensity of muscular catabolism. Considered together, these parameters describe not only renal function but also reveal a general metabolic immaturity that may render the retina more vulnerable to the onset of neovascularization [32,33].

Nutritional, hepatic, and renal parameters can provide valuable insights into the body’s ability to sustain physiological angiogenesis and maintain the metabolic balance required for retinal development [34]. Based on these premises, analyzing the relationship between nutrition, hepatic function, and renal function becomes essential for understanding the mechanisms involved in ROP and for identifying potential clinical and biological predictors of disease severity. Although there are data suggesting the involvement of nutrition and metabolic factors in the progression of this pathology, their predictive value for disease risk and severity remains insufficiently documented [12]. The present study aims to analyze the relationship between nutrition, hepatic and renal function, and ROP stages, evaluating whether serum levels of total protein, blood glucose, transaminases, urea and creatinine may serve as clinical–biological biomarkers with predictive value for disease risk and severity.

## 2. Materials and Methods

Design: This retrospective study included 140 preterm infants born between January 2021 and December 2024 in the neonatal intensive care units of “Sf Ioan” Emergency Clinical Hospital for Children and “Sf Andrei” County Emergency Clinical Hospital in Galați, Romania. Although systemic risk factors are similar, ROP may evolve differently between the two eyes of the same premature infant. For this reason, the diagnosis was established and analyzed separately for each eye, in order to accurately capture the variability of the disease. Under these conditions, 280 eyes from the 140 preterm infants were included, providing a more solid statistical basis and allowing for a more rigorous interpretation of the results. Among them, 150 eyes (53.57%) presented ROP, while 130 (46.43%) showed no signs of this pathology. At the infant level, ROP was diagnosed in 77/140 infants (55.0%), being bilateral in 73 infants (52.14%) and unilateral in 4 infants (2.86%), with all unilateral cases classified as stage 1.

Inclusion criteria:

All preterm infants who cumulatively met the following conditions were included in the study: birth weight ≤ 2000 g, gestational age ≤ 34 weeks, availability of a sufficient number of laboratory tests performed during hospitalization, availability of data regarding feeding during the hospital stay, and complete ophthalmological screening for ROP.

Exclusion criteria:

Preterm infants with major congenital malformations, severe hematological or infectious diseases that could have influenced biological parameters, as well as those with missing essential medical data, particularly regarding biological monitoring, nutritional information, or ophthalmological evaluations were excluded from the analysis.

Data collection and analyzed variables:

Data were retrospectively obtained from medical records, laboratory records, and ophthalmological reports corresponding to the hospitalization period. Subsequently, all information was centralized into an anonymized electronic database, with each case coded numerically to ensure confidentiality.

The following categories of variables were analyzed:

- Demographic data: gestational age (GA), birth weight (BW), and sex;
- Nutritional parameters: type of feeding during hospitalization and at discharge (breast milk, formula, or mixed feeding), timing of enteral feeding initiation, and duration of parenteral nutrition;
- Serum metabolic parameters: levels of aspartate aminotransferase (AST), alanine aminotransferase (ALT), total protein, blood glucose, urea and creatinine, collected sequentially on days 1, 3, 5, 7, 14, 21, and 28 of life, depending on the duration of hospitalization. IGF-1 and VEGF measurements were not available in the medical records and were therefore not included among the analyzed variables.
- Ophthalmological evaluation: examinations were performed separately for each eye, recording the presence or absence of ROP, the highest stage observed, the lesion location according to the International Classification of Retinopathy of Prematurity (ICROP), and any therapeutic recommendations.

Evaluation of nutritional parameters. Feeding data were recorded for each infant throughout hospitalization and at discharge. Enteral feeding refers to any gastrointestinal nutrient intake (oral or via feeding tube), including mother’s own milk, infant formula, or both. Parenteral nutrition (PN) refers to postnatal intravenous nutritional support administered during hospitalization when enteral intake is not possible or remains insufficient. In this study, PN was recorded only after birth; prenatal maternal nutritional interventions were not assessed.

Feeding was categorized as natural (mother’s own milk), artificial (infant formula), or mixed (combination of mother’s own milk and formula during the assessed period). Information was retrieved from patient charts and ward records updated daily by the medical team. Feeding categories were based on the documented type of milk administered in routine clinical records, while detailed nutritional composition (e.g., fortification or caloric density) was not separately quantified in this study.

Data were extracted from patient charts and ward records, which were updated on a daily basis by the medical team.

Evaluation of metabolic parameters: Biochemical analyses were performed sequentially, in accordance with institutional protocols, on days 1, 3, 5, 7, 14, 21, and 28 of life. The distribution of eyes according to monitoring duration was as follows: data were available up to day 28 for 222 eyes, up to day 21 for 26 eyes, up to day 14 for 14 eyes, and up to day 7 for 18 eyes. All biochemical determinations were performed using periodically calibrated automated analyzers, with no methodological changes during the study period.

Ophthalmological screening: All eyes included in the study were examined for ROP by indirect ophthalmoscopy using a 20D lens, after pupil dilation with 0.5% tropicamide and 2.5% phenylephrine. The first examination was performed at a postnatal age of 4 weeks or at 31–33 weeks of postmenstrual age, according to the screening protocol. Retinal changes were classified based on ICROP criteria, and the schedule of follow-up examinations was established according to the stage and location of the observed lesions. Monitoring continued until complete retinal vascularization or full disease regression was achieved. Cases meeting the criteria for Type 1 ROP were referred for treatment initiation in specialized centers, in accordance with national and international guidelines.

Statistical analysis: Statistical processing was performed using IBM SPSS Statistics version 29.0. Numerical variables were described as mean ± standard deviation or as median and interquartile range, depending on their distribution. Normality was assessed using the Shapiro–Wilk test and visual inspection of Q–Q plots. As most continuous variables deviated from a normal distribution, comparisons of parameters among groups defined by ROP stages were made using the Kruskal–Wallis test. Associations between categorical variables (type of feeding, blood glucose level) and ROP stages were analyzed using Pearson’s Chi-square test. A *p* value < 0.05 was considered statistically significant.

Ethical approval: The study was conducted in accordance with the ethical principles outlined in the Declaration of Helsinki and was approved by the ethics committees of the following institutions: “Dunărea de Jos” University of Galați, Romania (approval no. 7284, 19 March 2024); “Sf Ioan” Emergency Clinical Hospital for Children, Galați, Romania (approval no. 2844, 29 February 2024); the Ethics Committee of the Medical College of Galati (approval no. 138, 9 February 2024); and “Sf Andrei” County Emergency Clinical Hospital, Galați, Romania (approval no. 2955, 6 February 2024). Written informed consent was obtained from all participants before inclusion in the study, and all data were anonymized prior to statistical processing to ensure patient confidentiality throughout the analysis.

## 3. Results

### 3.1. General Characteristics of the Study Population

**Figure 1:**
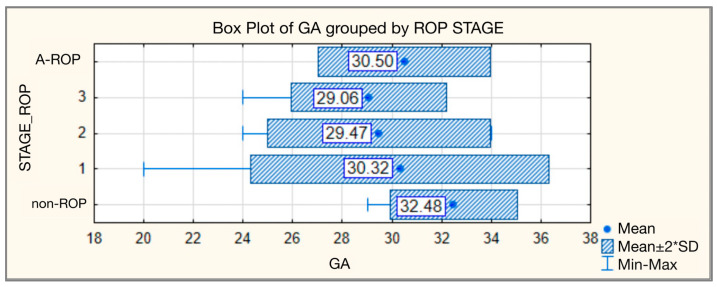
Gestational age—comparative analysis according to ROP stages.

The study included 140 preterm infants. Considering that a preterm infant, although exposed to the same risk factors, may present different ROP stages in each eye, the analysis was performed separately for each eye. In total, 280 eyes were examined, of which 150 (53.57%) presented ROP, while 130 (46.43%) showed no signs of this pathology. The highest gestational age was observed in the non-ROP group, with a mean of 32.48 ± 1.283 weeks and a median of 33.00. GA decreased proportionally with increasing ROP stage, reaching a mean of 29.06 ± 1.569 weeks and a median of 30.00 for stage 3. The A-ROP group had a mean gestational age of 30.50 ± 1.732 weeks and a median of 30.50. All these differences were statistically significant (Table 1, Figure 1).

**Figure 2:**
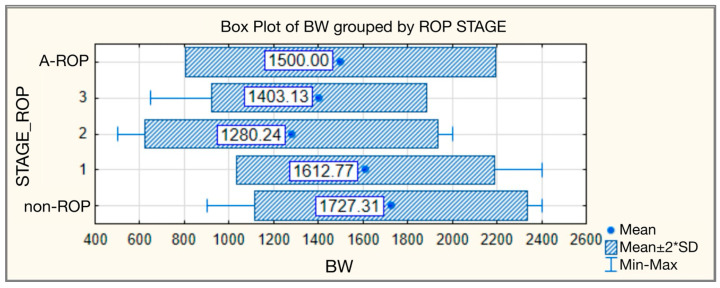
Birth weight—comparative analysis according to ROP stages.

Significant statistical differences were also observed in birth weight according to ROP stages. The highest birth weight (BW) values were found in the non-ROP group, with a mean of 1727.31 ± 305.195 g and a median of 1700.00 g, while the lowest values were recorded in the stage 2 ROP group, with a mean of 1280.24 ± 328.262 g and a median of 1300.00 g. The A-ROP group had a mean birth weight of 1500.00 ± 346.410 g and a median of 1500.00 g (Table 2, Figure 2).

### 3.2. Nutritional Parameters and Their Influence on the Progression of ROP

#### 3.2.1. Timing of Enteral Feeding Initiation

**Figure 3:**
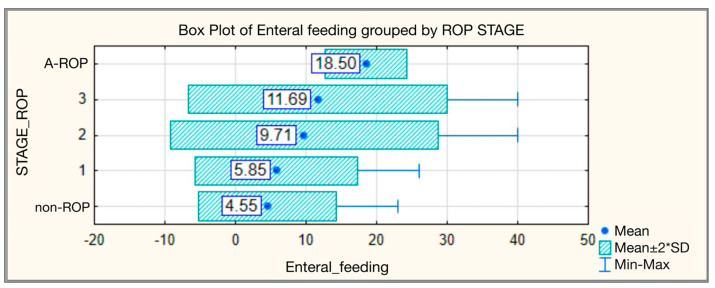
Enteral feeding—comparative analysis according to ROP stages.

On average, enteral feeding was initiated considerably earlier in the non-ROP group, at 4.55 ± 4.863 days (median 3.00), compared with the stage 3 ROP group, where feeding began at 11.69 ± 9.185 days (median 12.00). In the A-ROP group, enteral nutrition was introduced even later, typically between days 16 and 21, with statistically significant differences observed across the groups (Table 3, Figure 3).

#### 3.2.2. Duration of Parenteral Nutrition

**Figure 4:**
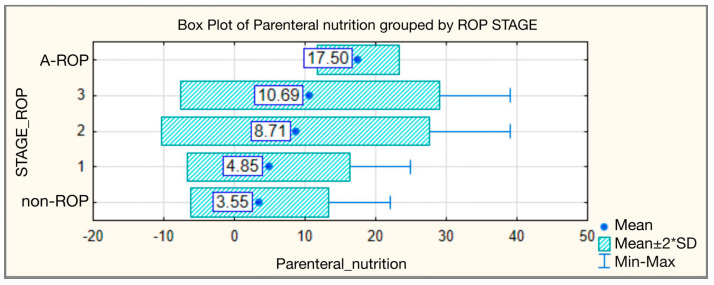
Parenteral nutrition—comparative analysis according to ROP stages.

The longest duration of total parenteral nutrition was observed in the A-ROP group, with a mean of 17.50 ± 2.887 days and a median of 17.50 (range 15–20 days). In infants with stage 3 ROP, the duration was significantly higher than in the non-ROP group, with a mean of 10.69 ± 9.185 days and a median of 11.00, compared to only 3.55 ± 4.863 days and a median of 2.00 in the non-ROP group. The differences between groups were highly statistically significant (Table 4, Figure 4).

#### 3.2.3. Feeding Regimen During Hospitalization and at Discharge

**Figure 5:**
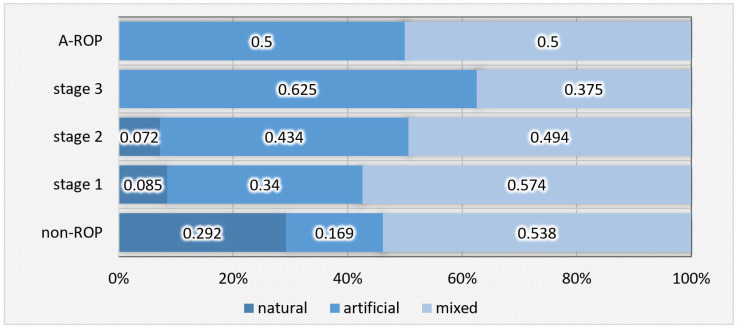
Distribution of newborns according to the type of hospital feeding in relation to ROP stages.

Statistically significant differences were also found in the type of feeding, both during hospitalization and at discharge. Natural feeding was present in nearly one-third of the newborns in the non-ROP group but was rare or absent in the ROP groups. In contrast, artificial feeding predominated in stage 3 ROP, while in stage 2 ROP and A-ROP, similar proportions of infants received artificial and mixed feeding (Table 5, Figure 5).

**Figure 6:**
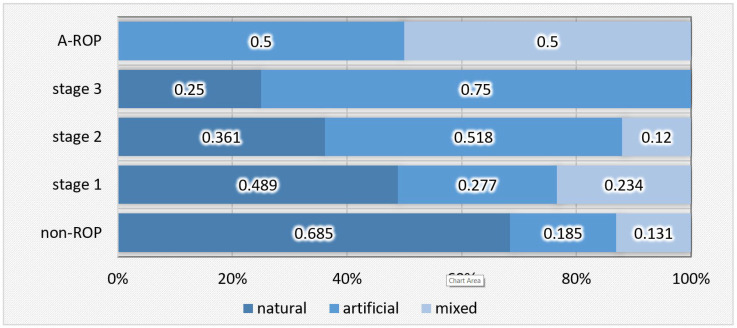
Distribution of newborns according to the type of feeding at discharge in relation to ROP stages.

At discharge, natural feeding predominated in the non-ROP group (68.5%), decreasing progressively with increasing ROP severity and being absent in the A-ROP group. Artificial feeding was predominant in stage 3 (75.0%) and frequent in stage 2 (51.8%), with statistically significant differences observed between the groups (Table 6, Figure 6).

### 3.3. Serum Metabolic Parameters and Their Association with ROP Severity

#### 3.3.1. Serum Transaminases (AST and ALT) 

**Figure 7:**
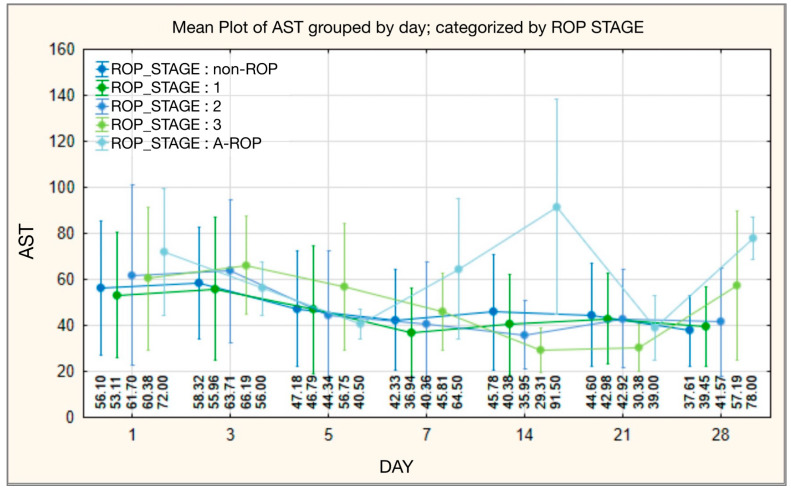
AST—temporal evolution of values in relation to ROP stages.

On day 1, AST values were lower in the non-ROP and stage 1 groups, with the lowest levels in stage 1 (median 45.00), and no significant differences across stages (Table 7). Across follow-up, AST values tended to be higher with increasing ROP severity, reaching the highest levels in the A-ROP group. By day 7, between-stage differences became statistically significant (*p* = 0.037), with the highest median in A-ROP (median 64.50) and the lowest median in stage 1 (median 33.00). This pattern was most pronounced on day 14 (*p* < 0.001) and remained significant by day 28 (*p* = 0.007), when AST again peaked in A-ROP (median 78.00) and was lowest in the non-ROP group (median 32.00) (Table 7, Figure 7).

**Figure 8:**
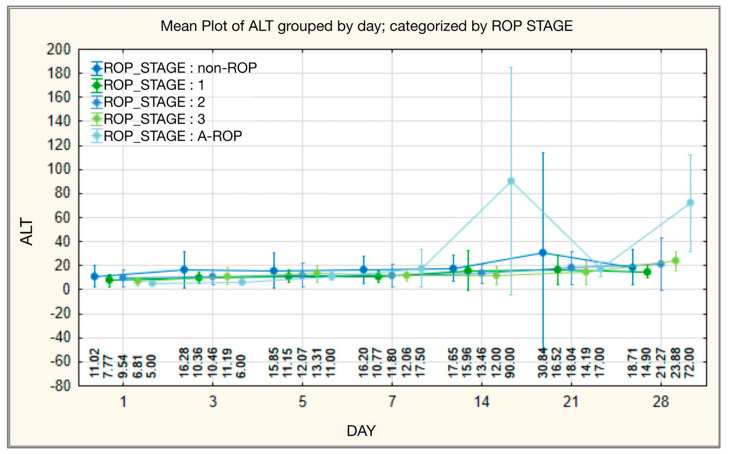
ALT—temporal evolution of values in relation to ROP stages.

Initially, ALT values did not differ significantly between ROP stages, although the highest levels were recorded in the non-ROP group (mean 11.0208 ± 8.95646; median 8.0000) and the lowest in the A-ROP group (mean 5.0000 ± 0.0000; median 5.0000). On day 3 of monitoring, the differences reached statistical significance, with higher values in the non-ROP group (16.2821 ± 15.35364; median 12.0000) and lower in the A-ROP group (6.0000 ± 1.15470; median 6.0000). The differences between groups diminished on day 5; however, from day 7 onward, ALT values in the A-ROP group rose progressively and became significantly higher than in the other groups. This trend persisted on days 14 and 28, when the A-ROP group showed the highest values (mean 72.0000 ± 40.41533; median 72.0000), while the ROP stage 0 and 1 groups recorded lower levels than those observed in stages 2 and 3 (Table 8, Figure 8).

#### 3.3.2. Total Protein

**Figure 9:**
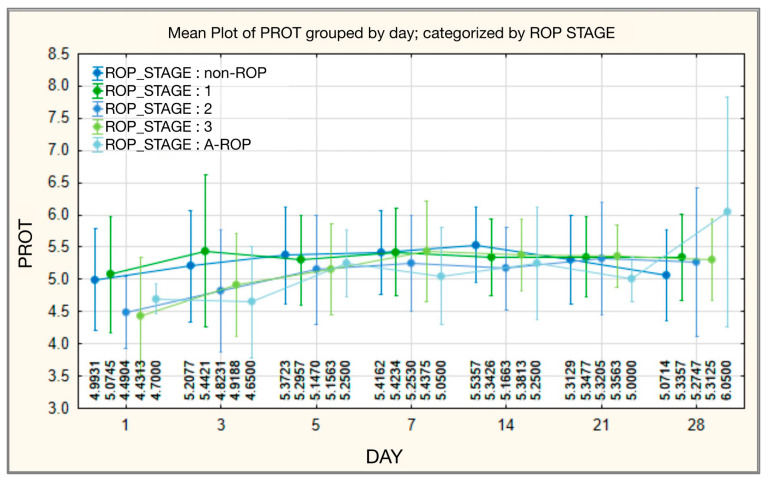
Total protein—temporal evolution of values in relation to ROP stages.

Initially, total protein (PROT) values showed highly significant statistical differences among ROP stages, being lowest in the stage 3 ROP group (mean 4.4313 ± 0.91922; median 4.2500), while the A-ROP group had intermediate values (mean 4.7000 ± 0.23094; median 4.7000). Between days 3 and 14, the values were slightly higher in the non-ROP and stage 1 ROP groups compared to stages 2 and 3, while remaining lower in the A-ROP group. At the end of the period (day 28), the highest values were recorded in the A-ROP group (mean 6.0500 ± 1.78979; median 6.0500), clearly exceeding those of the other groups (Table 9, Figure 9).

#### 3.3.3. Blood Glucose

**Figure 10:**
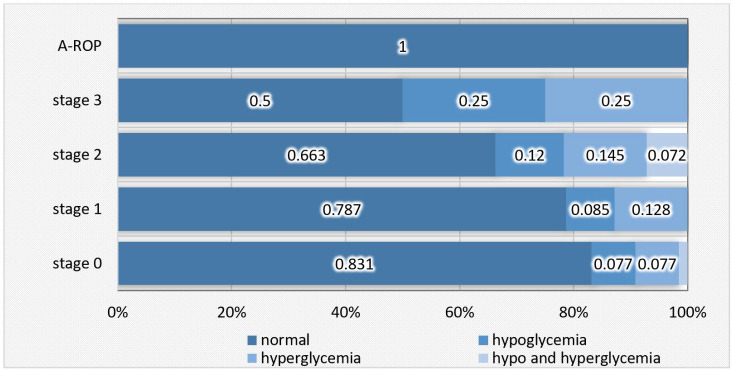
Distribution of newborns according to blood glucose levels in relation to ROP stages.

In the A-ROP group, as well as in approximately 80% of cases from the non-ROP and stage 1 ROP groups, blood glucose levels were within the normal range. The proportion of normal values was significantly lower in stage 2 (66.3%) and stage 3 (50.0%), where cases of both hypo- and hyperglycemia were observed more frequently (Table 10, Figure 10).

#### 3.3.4. Serum Urea

**Figure 11:**
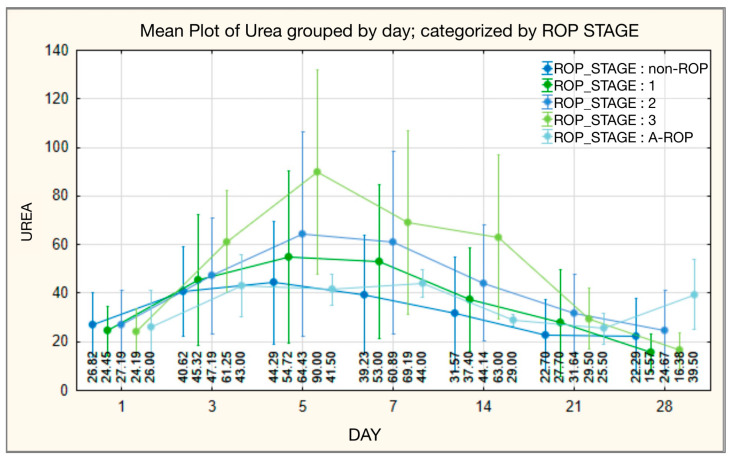
Serum urea—temporal evolution of values in relation to ROP stages.

Although initial urea values did not differ significantly among ROP stages, from the third day of monitoring onward they diverged significantly, a pattern that persisted until the end of the observation period. On day 3, values increased progressively with disease severity, with the lowest levels in stage 0 (mean 40.62 ± 18.283; median 38.00) and the highest in stage 3 (mean 61.25 ± 21.174; median 64.00), while the A-ROP group showed intermediate levels (mean 43.00 ± 12.702; median 43.00). The same trend was observed on days 5, 7, 14, and 21, with a gradual decline in values within the A-ROP group. At the end of the period (day 28), the A-ROP group again showed the highest values (mean 39.50 ± 14.434; median 39.50), whereas the lowest were recorded in stage 1 (mean 15.57 ± 7.828; median 13.00) (Table 11, Figure 11).

#### 3.3.5. Serum Creatinine

**Figure 12:**
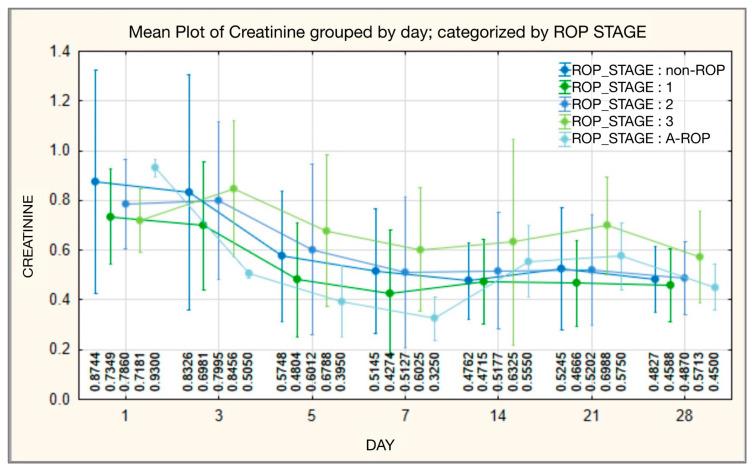
Serum creatinine—temporal evolution of values in relation to ROP stages.

At baseline (day 1), serum creatinine differed significantly across ROP stages (*p* = 0.010), with the highest median in the A-ROP group (median 0.9300) and the lowest median in stage 3 (median 0.7000). On day 3, differences across stages remained significant (*p* = 0.017), and the A-ROP group showed the lowest values (median 0.5050). On day 5, between-stage differences were not statistically significant (*p* = 0.093), although higher medians were observed in stages 2–3 compared with stages 0–1. By day 7, the overall comparison across stages was significant again (*p* = 0.009), with the highest median in stage 3 (median 0.5650). On day 14, creatinine values were comparable across stages (*p* = 0.243). On day 21, differences were significant (*p* = 0.004), with the highest median observed in stage 3 (median 0.7600). By day 28, between-stage differences were no longer significant (*p* = 0.383), and median values clustered within a narrow range across groups (Table 12, Figure 12).

## 4. Discussion

Enteral nutrition represents an essential element in the care of preterm infants, playing a central role not only in supporting overall growth and development but also in the maturation of sensitive structures such as the retina. Unlike parenteral nutrition, which ensures basic survival and growth, enteral feeding provides biological factors with trophic and metabolic effects that cannot be fully replaced [35,36]. Delayed introduction of enteral nutrition may increase the vulnerability of preterm infants due to the absence of specific stimuli, with the potential to influence the physiological process of retinal vascularization [11].

Our results show a significant association between the timing of enteral feeding initiation and the risk of developing ROP. In the non-ROP group, enteral feeding was initiated early, at an average of 4.55 ± 4.863 days (median 3), compared with the advanced stages of the disease, where initiation occurred much later: 11.69 ± 9.185 days (median 12) in stage 3 ROP and approximately 18.5 days in A-ROP. A progressive pattern can thus be observed, with the initiation of enteral feeding being increasingly delayed in patients with more severe forms of ROP (H = 43.654, *p* < 0.001). This trend is also supported by the literature, which reports that in infants with advanced ROP, enteral feeding was introduced significantly later than in control groups or in those with milder forms, confirming the relationship between delayed initiation and disease progression [37]. Moreover, the multicenter G-ROP study, conducted on more than 7400 preterm infants, demonstrated that the administration of any type of enteral feeding within the first six weeks of life was associated with a significant reduction in the risk of ROP (adjusted OR 0.50–0.74) and severe forms (adjusted OR 0.43–0.59), confirming an independent protective effect, irrespective of gestational age or birth weight [38].

Parenteral nutrition is indispensable during the first days of life, when enteral feeding cannot fully meet the infant’s energy and nutritional requirements. Although it ensures basic survival and growth, prolonged use of PN may reflect the clinical fragility of the preterm infant and can indirectly influence the evolution of complications associated with prematurity [39]. Recent publications indicate that an aggressive nutritional strategy, consisting of the early initiation of both intravenous and enteral nutrition followed by a rapid transition to full enteral feeding is safe and effective [40,41]. However, several studies have reported that a longer duration of PN was associated with severe forms of ROP [42,43]. Moreover, incorporating parenteral nutrition duration exceeding 14 days as a variable in the DIGI-ROP clinical decision-support algorithm improved its predictive performance, allowing for more accurate identification of preterm infants at increased risk of ROP and optimization of monitoring strategies [44,45]

Our analysis confirms these observations, highlighting a statistically significant difference (H = 43.654, *p* < 0.001) in parenteral nutrition duration among the groups. In the non-ROP group, the duration was shorter, with a mean of 3.55 ± 4.863 days and a median of 2 days. This increased progressively with disease severity: 4.85 ± 5.748 days (median 2) in stage 1, 8.71 ± 9.473 days (median 4) in stage 2, and 10.69 ± 9.185 days (median 11) in stage 3. The highest values were recorded in infants with A-ROP, where parenteral nutrition duration reached 17.50 ± 2.887 days (median 17.5; range 15–20).

The feeding strategy administered to preterm infants represents an important determinant of clinical evolution, with potential implications for the risk of developing ROP. Breast milk is considered the gold standard, providing growth factors, bioactive compounds and partial protection against oxidative stress, which may reduce the risk of disease onset and progression [11]. Although some studies have not identified a significant association between breast milk and ROP, the majority of publications confirm its protective and metabolic benefits [46,47]. Compared with formula feeding, maternal breast milk was associated with a significantly lower prevalence of stage 3 proliferative forms (5.6% vs. 14%), suggesting a specific protective effect against severe forms [48,49]. Similar results were reported by Manzoni et al., who observed a lower prevalence of ROP among breastfed infants compared with those fed with formula (3.5% vs. 15.8%) [50]. Several studies have confirmed these benefits, showing that exclusive feeding with human milk was associated with a significant reduction in the incidence of ROP (5.2% vs. 9%, *p* = 0.003) [51], while a meta-analysis demonstrated that even partial amounts of human milk could provide protection against the disease [52]. Other clinical observations reported higher IGF-1 levels in infants fed with breast milk, which may explain this protective role [34,53].

The observations from our study are consistent with findings from the literature, and statistical analysis revealed significant differences regarding the type of feeding both during hospitalization (Chi-Square = 40.716; *p* < 0.001) and at discharge (Chi-Square = 50.222; *p* < 0.001). During hospitalization, natural feeding was present in nearly one-third of newborns in the non-ROP group (29.2%) but decreased significantly with increasing disease severity, being absent in patients with stage 3 ROP and A-ROP. In these groups, artificial feeding predominated (62.5% in stage 3 and 50% in A-ROP), while in stages 1 and 2, proportions were divided between artificial and mixed feeding. At discharge, this trend became more pronounced: natural feeding predominated in the non-ROP group (68.5%) but decreased progressively with disease severity, reaching only 25% in stage 3 and being absent in A-ROP. Conversely, artificial feeding was recorded in 75% of infants with stage 3 ROP and in half of those with A-ROP.

These findings emphasize the importance of the type of feeding in the progression and severity of ROP, supporting the protective role of breast milk, as demonstrated both in the literature and in the data obtained in this study. In the context of ROP risk, promoting natural feeding whenever possible and limiting the use of formula milk could represent valuable clinical strategies for reducing the incidence and severity of the disease [54,55].

The liver is the main systemic source of IGF-1, and changes in biochemical hepatic parameters may be considered indirect markers of the maturity and functionality of this organ, exerting an indirect impact on circulating levels of this factor. Reduced IGF-1 levels have previously been associated with an increased risk of ROP onset and progression, suggesting that the analysis of transaminases and other hepatic parameters may provide relevant insights into the complex relationship between neonatal metabolic status and susceptibility to disease development [34,56,57].

The literature shows conflicting evidence concerning AST variations in ROP. Some studies reported no significant differences in AST between groups, whereas others described lower AST activity in infants with ROP, which may reflect metabolic adaptation rather than overt hepatic injury [25,34]. In our cohort, we observed a stage-related AST pattern over time. At baseline (day 1), AST values were lower in the non-ROP and stage 1 groups and highest in the A-ROP group (median 72.00). Differences across stages were most pronounced on day 14 (*p* < 0.001), and by day 28 AST remained highest in A-ROP (median 78.00) and lowest in non-ROP (median 32.00) (Table 7, Figure 7). Because AST is a non-specific marker influenced by hepatic immaturity and extrahepatic sources in preterm infants, these associations may reflect systemic metabolic stress and the overall burden of prematurity in infants with more severe ROP rather than a retina-specific mechanism.

Regarding ALT, the data available in the literature are limited. One study reported slightly higher ALT levels in infants with type 2 ROP compared with those with type 1 ROP and non-ROP, although without statistical significance [34]. Reference values also indicate that, in preterm infants, normal levels range between 6 and 38 U/L, with medians of 13–16 U/L depending on corrected gestational age [58]. In our analysis, ALT values did not show statistically significant differences between groups in the first days; however, from day 7, a progressive and statistically significant increase was observed in the A-ROP group (*p* = 0.001), a trend that persisted throughout the monitoring period. This evolution, characterized by persistently elevated levels in severe forms, suggests hepatic injury associated with increased oxidative stress and highlights a possible link between metabolic dysfunction and ROP severity.

Serum total protein and albumin levels play an essential role in maintaining the metabolic balance of preterm infants, being closely correlated with serum IGF-1 levels, a key factor for physiological retinal angiogenesis. Although most published studies have focused on albumin, this represents the main fraction of serum proteins, and its variations may indirectly reflect the overall protein status. Low albumin levels have been frequently associated with severe forms of ROP requiring treatment and with unfavorable neonatal outcomes [59,60,61]. Our results partially confirm these data, showing significant differences in total protein values between groups, with the lowest levels recorded in preterm infants with advanced forms of ROP. In the non-ROP group, proteins presented higher values during the first days (mean 4.99 ± 0.78; median 4.95), while in the A-ROP group, they were lower at baseline (mean 4.70 ± 0.23; median 4.70). Subsequently, a significant increase in levels was observed in the A-ROP group, probably related to the optimization of nutritional support, with values progressively approaching those of the other groups. This evolution suggests a relationship between protein status and ROP severity, emphasizing the influence of metabolic balance on disease progression.

Glucose is the main source of energy for the developing retina, being essential for photoreceptor function and physiological angiogenesis. During the neonatal period, maintaining stable glycemic homeostasis is essential, as both hypoglycemia and hyperglycemia can disrupt retinal vascular development [62,63,64]. Evidence from the literature indicates a significant association between neonatal hyperglycemia and an increased risk of ROP, particularly of severe forms. Serum glucose levels ≥ 150 mg/dL (8.3 mmol/L) have been correlated with an almost fourfold higher risk of developing ROP (95% CI 2.36–6.53) [55]. Elevated glucose levels in the first month of life have also been associated with more advanced disease stages and reduced serum IGF-1 levels, suggesting an imbalance between pro- and anti-angiogenic factors. The mechanisms involved include oxidative stress, inflammation, and reduced IGF-1 expression concomitant with VEGF activation [24,65]. In addition to hyperglycemia, fluctuations in glucose levels and hypoglycemic episodes may also contribute to instability in retinal vascular development. These variations, frequently observed during the neonatal period, can affect retinal metabolic and angiogenic processes, amplifying the vulnerability of immature vessels [66,67].

In line with these observations, our study results demonstrated a direct relationship between glycemic balance and ROP severity. Blood glucose values remained within normal limits in approximately 80% of newborns in the non-ROP and stage 1 groups and in all A-ROP cases, but this proportion decreased in stage 2 (66.3%) and stage 3 (50.0%), where hypo- and hyperglycemic episodes were more frequent (χ^2^ = 22.441; *p* = 0.033). These findings support the hypothesis that instability in blood glucose levels during the neonatal period may contribute to ROP progression by disrupting mechanisms dependent on IGF-1 and VEGF.

Serum urea reflects both hepatic protein metabolism and renal function, and in preterm infants, its variations may represent an indirect marker of metabolic stress Neonatal renal dysfunction is explained by the reduced number of nephrons and low glomerular density, consequences of interrupted glomerulogenesis [68,69]. Oxidative stress, recognized as a common mechanism involved in prematurity-associated conditions, affects both the retina and the kidneys, contributing to endothelial injury and the angiogenic imbalances characteristic of ROP [70,71]. Data from the literature have shown higher serum urea levels in patients with ROP, particularly in severe forms. In one study, the mean serum urea level was 50.08 ± 67.73 mg/dL in infants with type 1 ROP, compared with 26.18 ± 19.14 mg/dL in the control group (*p* = 0.020), these findings being interpreted as a possible indicator of retinal endothelial injury [34].

Consistent with these findings, our results showed significant differences in urea values starting from day 3 of monitoring and continuing until the end, with levels increasing progressively according to disease severity. Higher values were observed in the more advanced stages, particularly stage 3 (day 3: mean 61.25 ± 21.174; median 64.00; day 14: mean 63.00 ± 33.754; median 52.00), while lower values were recorded in the non-ROP group (day 3: mean 40.62 ± 18.283; median 38.00; day 14: mean 31.57 ± 23.351; median 23.00). In the A-ROP group, urea values were 43.00 ± 12.702 (median 43.00) on day 3 and 29.00 ± 2.309 (median 29.00) on day 14. Toward the end of the monitoring period, although values decreased across all groups, the differences persisted, with higher levels in severe ROP cases (*p* < 0.05). Our data support the hypothesis that elevated urea values are involved in the pathophysiology of ROP and may contribute to the development of more severe disease forms, having potential value in predicting ROP risk.

Serum creatinine is a widely used indicator of renal function; however, in preterm infants, its levels reflect not only glomerular filtration but also biological immaturity, muscle mass, and early postnatal adaptation. Therefore, creatinine fluctuations in this population may capture systemic metabolic adjustment to extrauterine life rather than overt renal dysfunction [72,73]. Clinical studies published to date have reported variable results. In a recent study, mean creatinine values were higher in preterm infants with severe ROP (0.71 mg/dL) compared with those with stage 1 and 2 ROP (0.65 mg/dL), although the difference did not reach statistical significance (*p* = 0.21) [28]. Other studies have suggested an indirect role for this parameter, noting that the urinary NT-proBNP/creatinine ratio was associated with an increased risk of severe ROP, while lower creatinine levels were proposed as a potential metabolic biomarker of ROP [74,75].

In our cohort, creatinine differed across ROP stages primarily in the early postnatal period. On day 1, between-stage differences were significant (*p* = 0.010), with the highest median in the A-ROP group (median 0.9300). On day 3, differences remained significant (*p* = 0.017), and the A-ROP group showed the lowest values (median 0.5050). Additional significant between-stage differences were observed on days 7 (*p* = 0.009) and 21 (*p* = 0.004), whereas creatinine values were comparable across stages on days 14 and 28 (Table 12, Figure 12). Overall, this pattern suggests that creatinine dynamics may reflect early systemic adaptation in infants with different ROP trajectories, and they did not remain consistently different at later time points.

These findings suggest that, although serum creatinine levels are not a specific biomarker for ROP, their distinct dynamics among subgroups and correlations with other metabolic parameters may reflect different degrees of renal immaturity and metabolic adaptation, aspects relevant to disease progression. Overall, these observations indicate that variations in creatinine levels may mirror the degree of renal immaturity and the metabolic imbalance associated with disease severity.

This study has several limitations that should be acknowledged. We did not perform multivariable analyses; therefore, the associations observed between nutritional and metabolic parameters and ROP severity may be influenced by the degree of prematurity. Lower gestational age and birth weight are typically associated with a longer NICU stay, greater oxygen exposure, and a later initiation of enteral feeding, factors that may affect both the biochemical profile and ROP stage. Prospective studies and multivariable models are needed to delineate independent effects.

## 5. Conclusions

The findings of our study highlight how closely nutritional and metabolic balance are linked to the progression of retinopathy of prematurity. Both the timing of enteral feeding initiation and the type of nutrition administered influenced metabolic stability and appeared to support physiological retinal angiogenesis. Early initiation of enteral feeding, together with natural feeding, was associated with milder forms of ROP, suggesting that timely nutritional interventions may help limit disease progression.

The assessment of nutritional, hepatic and renal parameters revealed a close association between the level of metabolic maturity and the severity of ROP. Higher concentrations of transaminases, urea and creatinine, combined with changes in total protein levels and glycemic fluctuations, were linked to more advanced stages of the disease, indicating the presence of systemic metabolic dysfunction. These alterations may reflect the hepatic and renal vulnerability of preterm infants and could serve as potential predictive biomarkers for disease evolution, offering practical value for risk stratification and early clinical monitoring. By analyzing these parameters, the study offers an original contribution to the understanding of the mechanisms involved in ROP and supports the need for a multidisciplinary approach that integrates metabolic and nutritional assessment into neonatal screening and monitoring programs. Early identification of preterm infants at higher risk could enable the implementation of personalized preventive strategies and improve the use of medical resources, allowing for their more efficient allocation to the cases where early intervention can have the greatest impact.

## Figures and Tables

**Table 1:** Gestational age—comparative analysis according to ROP stages.

Gestational Age									
ROP Stage	N	Mean	Standard Deviation	Min	Max	Median	IQR		Kruskal–Wallis Test
							25th	75th	
non-ROP	130	32.48	1.283	29	34	33.00	31.75	34.00	H = 114.754
1	47	30.32	3.008	20	34	31.00	29.00	32.00	*p* < 0.001
2	83	29.47	2.243	24	34	30.00	28.00	31.00	
3	16	29.06	1.569	24	30	30.00	28.25	30.00	
A-ROP	4	30.50	1.732	29	32	30.50	29.00	32.00	
Total	280	31.00	2.430	20	34	31.00	30.00	33.00	

**Table 2:** Birth weight—comparative analysis according to ROP stages.

Birth Weight									
ROP Stage	N	Mean	Standard Deviation	Min	Max	Median	IQR		Kruskal–Wallis Test
							25th	75th	
non-ROP	130	1727.31	305.195	900	2400	1700.00	1537.50	1900.00	H = 84.835
1	47	1612.77	288.826	1100	2400	1600.00	1400.00	1800.00	*p* < 0.001
2	83	1280.24	328.262	500	2000	1300.00	1100.00	1500.00	
3	16	1403.13	240.464	650	1650	1425.00	1300.00	1587.50	
A-ROP	4	1500.00	346.410	1200	1800	1500.00	1200.00	1800.00	
Total	280	1553.79	362.090	500	2400	1600.00	1300.00	1800.00	

**Table 3:** Enteral feeding—comparative analysis according to ROP stages.

Enteral Feeding—Day of Initiation									
ROP Stage	N	Mean	Standard Deviation	Min	Max	Median	IQR		Kruskal–Wallis Test
							25th	75th	
non-ROP	130	4.55	4.863	1	23	3.00	1.00	6.00	H = 43.654
1	47	5.85	5.748	1	26	3.00	2.00	8.00	*p* < 0.001
2	83	9.71	9.473	1	40	5.00	3.00	14.00	
3	16	11.69	9.185	4	40	12.00	4.00	16.00	
A-ROP	4	18.50	2.887	16	21	18.50	16.00	21.00	
Total	280	6.91	7.465	1	40	4.00	2.00	10.00	

**Table 4:** Parenteral nutrition—comparative analysis according to ROP stages.

Duration of Parenteral Nutrition									
ROP Stage	N	Mean	Standard Deviation	Min	Max	Median	IQR		Kruskal–Wallis Test
							25th	75th	
non-ROP	130	3.55	4.863	0	22	2.00	0.00	5.00	H = 43.654
1	47	4.85	5.748	0	25	2.00	1.00	7.00	*p* < 0.001
2	83	8.71	9.473	0	39	4.00	2.00	13.00	
3	16	10.69	9.185	3	39	11.00	3.00	15.00	
A-ROP	4	17.50	2.887	15	20	17.50	15.00	20.00	
Total	280	5.91	7.465	0	39	3.00	1.00	9.00	

**Table 5:** Distribution of newborns according to the type of hospital feeding in relation to ROP stages.

		ROP Stage										Total	
		Non-ROP		1		2		3		A-ROP			
		N	%	N	%	N	%	N	%	N	%	N	%
		Pearson Chi-Square = 40.716/*p* < 0.001											
Type of feeding during hospitalization	natural	38	29.2%	4	8.5%	6	7.2%					48	17.1%
	artificial	22	16.9%	16	34.0%	36	43.4%	10	62.5%	2	50.0%	86	30.7%
	mixed	70	53.8%	27	57.4%	41	49.4%	6	37.5%	2	50.0%	146	52.1%
Total		130	100.0%	47	100.0%	83	100.0%	16	100.0%	4	100.0%	280	100.0%

**Table 6:** Distribution of newborns according to the type of feeding at discharge in relation to ROP stages.

		ROP Stage										Total	
		Non-ROP		1		2		3		A-ROP			
		N	%	N	%	N	%	N	%	N	%	N	%
		Pearson Chi-Square = 50.222/*p* < 0.001											
Type of feeding at hospital discharge	natural	89	68.5%	23	48.9%	30	36.1%	4	25.0%			146	52.1%
	artificial	24	18.5%	13	27.7%	43	51.8%	12	75.0%	2	50.0%	94	33.6%
	mixed	17	13.1%	11	23.4%	10	12.0%			2	50.0%	40	14.3%
Total		130	100.0%	47	100.0%	83	100.0%	16	100.0%	4	100.0%	280	100.0%

**Table 7:** AST—temporal evolution of values in relation to ROP stages.

ROP Stage	N	Mean	Standard Deviation	Min	Max	Median	IQR		Kruskal–Wallis Test
							25th	75th	
AST 1									
non-ROP	130	56.10	29.312	20	190	48.50	34.50	72.00	H = 3.326
1	47	53.11	27.286	25	112	45.00	30.00	74.00	*p* = 0.505
2	83	61.70	39.147	15	197	49.00	38.00	67.00	
3	16	60.38	31.012	27	113	56.00	31.00	78.00	
A-ROP	4	72.00	27.713	48	96	72.00	48.00	96.00	
Total	280	57.73	32.299	15	197	49.00	34.25	71.75	
AST 3									
non-ROP	130	58.32	24.396	11	120	52.00	43.00	78.00	H = 5.032
1	47	55.96	31.203	15	153	46.00	30.00	78.00	*p* = 0.284
2	83	63.71	31.003	15	146	56.00	38.00	83.00	
3	16	66.19	21.495	27	94	67.00	50.00	83.00	
A-ROP	4	56.00	11.547	46	66	56.00	46.00	66.00	
Total	280	59.94	27.479	11	153	53.00	39.25	80.00	
AST 5									
non-ROP	130	47.18	25.112	12	121	39.50	29.00	62.00	H = 5.395
1	47	46.79	27.897	13	111	37.00	26.00	72.00	*p* = 0.249
2	83	44.34	28.217	18	125	34.00	24.00	56.00	
3	16	56.75	27.382	22	113	58.00	32.00	70.00	
A-ROP	4	40.50	6.351	35	46	40.50	35.00	46.00	
Total	280	46.72	26.510	12	125	37.50	26.00	61.75	
AST 7									
non-ROP	130	42.33	21.838	10	120	34.00	25.75	50.50	H = 10.215
1	47	36.94	19.409	13	98	33.00	23.00	43.00	*p* = 0.037
2	83	40.36	27.488	15	156	32.00	25.00	43.00	
3	16	45.81	16.829	26	70	39.00	28.50	62.00	
A-ROP	4	64.50	30.600	38	91	64.50	38.00	91.00	
Total	280	41.36	23.299	10	156	34.00	25.00	50.00	
AST 14									
non-ROP	112	45.78	24.952	16	136	41.50	29.00	53.50	H = 22.273
1	47	40.38	22.018	12	98	31.00	27.00	49.00	*p* < 0.001
2	83	35.95	15.095	15	74	31.00	25.00	47.00	
3	16	29.31	9.844	18	47	26.50	24.00	38.50	
A-ROP	4	91.50	46.765	51	132	91.50	51.00	132.00	
Total	262	41.39	22.755	12	136	34.00	26.00	50.00	
AST 21									
non-ROP	101	44.60	22.396	11	134	38.00	30.00	55.00	H = 7.508
1	44	42.98	19.578	13	85	37.00	28.00	51.00	*p* = 0.111
2	83	42.92	21.224	15	91	39.00	23.00	58.00	
3	16	30.38	10.197	20	53	28.00	22.25	38.00	
A-ROP	4	39.00	13.856	27	51	39.00	27.00	51.00	
Total	248	42.74	20.966	11	134	38.00	26.25	53.75	
AST 28									
non-ROP	77	37.61	15.327	17	85	32.00	26.00	46.00	H = 14.135
1	42	39.45	17.223	20	76	34.00	25.75	50.25	*p* = 0.007
2	83	41.57	23.542	15	121	33.00	24.00	53.00	
3	16	57.19	32.408	18	103	38.50	34.25	97.00	
A-ROP	4	78.00	9.238	70	86	78.00	70.00	86.00	
Total	222	41.58	21.492	15	121	34.00	26.00	53.00	

**Table 8:** ALT—temporal evolution of values in relation to ROP stages.

ROP Stage	N	Mean	Standard Deviation	Min	Max	Median	IQR		Kruskal–Wallis Test
							25th	75th	
ALT 1									
non-ROP	130	11.02	8.956	4	59	8.00	6.00	12.00	H = 7.115
1	47	7.77	5.053	2	26	7.00	4.00	9.00	*p* = 0.130
2	83	9.54	7.410	2	34	7.00	6.00	10.00	
3	16	6.81	3.124	2	12	6.00	5.00	9.00	
A-ROP	4	5.00	0.000	5	5	5.00	5.00	5.00	
Total	280	9.71	7.758	2	59	7.00	6.00	11.00	
ALT 3									
non-ROP	130	16.28	15.354	3	111	12.00	9.00	19.00	H = 26.709
1	47	10.36	4.734	4	25	9.00	7.00	12.00	*p* < 0.001
2	83	10.46	5.896	3	29	9.00	7.00	11.00	
3	16	11.19	7.432	4	28	8.00	6.00	15.00	
A-ROP	4	6.00	1.155	5	7	6.00	5.00	7.00	
Total	280	13.12	11.614	3	111	10.00	7.00	15.00	
ALT 5									
non-ROP	130	15.85	14.637	1	91	11.50	8.00	18.00	H = 9.025
1	47	11.15	4.982	5	22	9.00	7.00	15.00	*p* = 0.060
2	83	12.07	9.659	1	56	9.00	7.00	13.00	
3	16	13.31	6.887	6	24	9.00	6.75	19.75	
A-ROP	4	11.00	3.464	8	14	11.00	8.00	14.00	
Total	280	13.73	11.722	1	91	10.00	7.00	17.00	
ALT 7									
non-ROP	130	16.20	11.280	3	67	13.00	9.00	20.50	H = 17.793
1	47	10.77	4.997	4	20	10.00	8.00	14.00	*p* = 0.001
2	83	11.80	9.052	4	56	10.00	6.00	14.00	
3	16	12.06	5.079	6	21	10.00	9.00	16.25	
A-ROP	4	17.50	15.588	4	31	17.50	4.00	31.00	
Total	280	13.76	9.840	3	67	11.00	8.00	17.00	
ALT 14									
non-ROP	112	17.65	10.708	0	66	16.00	12.00	20.75	H = 17.677
1	47	15.96	16.559	0	89	11.00	8.00	19.00	*p* = 0.001
2	83	13.46	8.151	4	51	12.00	8.00	17.00	
3	16	12.00	7.465	6	32	8.00	6.25	16.00	
A-ROP	4	90.00	94.685	8	172	90.00	8.00	172.00	
Total	262	16.78	17.656	0	172	13.00	9.00	19.00	
ALT 21									
non-ROP	101	30.84	83.580	5	612	17.00	13.00	25.00	H = 14.001
1	44	16.52	11.913	3	62	12.50	11.00	19.00	*p* = 0.007
2	83	18.04	13.345	2	80	14.00	10.00	21.00	
3	16	14.19	10.021	2	34	9.00	7.50	22.75	
A-ROP	4	17.00	5.774	12	22	17.00	12.00	22.00	
Total	248	22.72	54.451	2	612	15.00	11.00	22.00	
ALT 28									
non-ROP	77	18.71	14.798	6	97	16.00	11.00	21.00	H = 21.701
1	42	14.90	5.304	4	25	15.00	11.00	20.00	*p* < 0.001
2	83	21.27	21.545	3	115	14.00	10.00	22.00	
3	16	23.88	8.082	4	34	24.00	20.00	30.50	
A-ROP	4	72.00	40.415	37	107	72.00	37.00	107.00	
Total	222	20.28	18.315	3	115	15.00	11.00	22.00	

**Table 9:** Total protein—temporal evolution of values in relation to ROP stages.

ROP Stage	N	Mean	Standard Deviation	Min	Max	Median	IQR		Kruskal–Wallis Test
							25th	75th	
PROT 1									
non-ROP	130	4.9931	0.78850	3.30	7.20	4.9500	4.4750	5.5000	H = 33.062
1	47	5.0745	0.90685	3.60	7.60	5.0000	4.5000	5.4000	*p* < 0.001
2	83	4.4904	0.55538	3.00	5.90	4.6000	4.1000	4.7000	
3	16	4.4313	0.91922	3.00	6.50	4.2500	4.0000	4.7000	
A-ROP	4	4.7000	0.23094	4.50	4.90	4.7000	4.5000	4.9000	
Total	280	4.8214	0.79106	3.00	7.60	4.7000	4.3000	5.2000	
PROT 3									
non-ROP	130	5.2077	0.86218	2.50	7.20	5.3000	4.7000	5.8000	H = 19.810
1	47	5.4421	1.17800	3.70	8.84	5.2000	4.5000	6.1000	*p* < 0.001
2	83	4.8231	0.94212	3.60	8.36	4.6000	4.1000	5.3000	
3	16	4.9188	0.79517	4.00	6.50	4.6000	4.4250	5.6000	
A-ROP	4	4.6500	0.86603	3.90	5.40	4.6500	3.9000	5.4000	
Total	280	5.1086	0.96336	2.50	8.84	5.1000	4.5000	5.6000	
PROT 5									
non-ROP	130	5.3723	0.75329	3.10	7.20	5.4000	4.9000	5.9000	H = 5.823
1	47	5.2957	0.69843	3.60	6.80	5.3000	5.0000	5.6000	*p* = 0.213
2	83	5.1470	0.83685	3.70	7.00	5.3000	4.5000	5.8000	
3	16	5.1563	0.71365	4.30	6.30	5.0500	4.5250	5.6000	
A-ROP	4	5.2500	0.51962	4.80	5.70	5.2500	4.8000	5.7000	
Total	280	5.2786	0.76751	3.10	7.20	5.3000	4.8000	5.8000	
PROT 7									
non-ROP	130	5.4162	0.65602	2.80	6.50	5.5000	4.9000	5.9250	H = 5.440
1	47	5.4234	0.67186	4.40	6.70	5.2000	5.0000	5.9000	*p* = 0.245
2	83	5.2530	0.74052	4.00	6.90	5.1000	4.6000	5.7000	
3	16	5.4375	0.78305	4.40	6.60	5.1000	4.8250	6.4500	
A-ROP	4	5.0500	0.75056	4.40	5.70	5.0500	4.4000	5.7000	
Total	280	5.3650	0.69343	2.80	6.90	5.3500	4.9000	5.9000	
PROT 14									
non-ROP	112	5.5357	0.58323	4.30	6.60	5.5000	5.1250	6.0750	H = 16.149
1	47	5.3426	0.59004	4.60	6.40	5.2000	4.8000	5.8000	*p* = 0.003
2	83	5.1663	0.64359	3.70	6.90	5.1000	4.8000	5.6000	
3	16	5.3813	0.55764	4.50	6.20	5.4000	4.9000	5.9500	
A-ROP	4	5.2500	0.86603	4.50	6.00	5.2500	4.5000	6.0000	
Total	262	5.3702	0.62290	3.70	6.90	5.4000	4.9000	5.9000	
PROT 21									
non-ROP	101	5.3129	0.68697	4.10	7.00	5.3000	4.7500	5.8000	H = 1.274
1	44	5.3477	0.62561	4.00	6.60	5.3000	5.0000	5.7000	*p* = 0.866
2	83	5.3205	0.87175	3.00	7.70	5.3000	4.7000	6.0000	
3	16	5.3563	0.48576	4.60	6.20	5.5000	5.0250	5.6000	
A-ROP	4	5.0000	0.34641	4.70	5.30	5.0000	4.7000	5.3000	
Total	248	5.3194	0.72745	3.00	7.70	5.3000	4.7250	5.7750	
PROT 28									
non-ROP	77	5.0714	0.70652	3.90	6.70	5.0000	4.5500	5.6000	H = 6.006
1	42	5.3357	0.67094	3.90	6.60	5.2000	4.8750	5.9000	*p* = 0.199
2	83	5.2747	1.15059	3.80	10.20	5.0000	4.5000	5.9000	
3	16	5.3125	0.63232	4.20	6.10	5.2000	4.9250	6.0000	
A-ROP	4	6.0500	1.78979	4.50	7.60	6.0500	4.5000	7.6000	
Total	222	5.2324	0.91722	3.80	10.20	5.0000	4.6000	5.7000	

**Table 10:** Distribution of newborns according to blood glucose levels in relation to ROP stages.

		ROP Stage										Total	
		Non-ROP		1		2		3		A-ROP			
		N	%	N	%	N	%	N	%	N	%	N	%
		Pearson Chi-Square = 22.441/*p* = 0.033											
Blood Glucose	normal	108	83.1%	37	78.7%	55	66.3%	8	50.0%	4	100.0%	212	75.7%
	hypoglycemia	10	7.7%	4	8.5%	10	12.0%	4	25.0%			28	10.0%
	hyperglycemia	10	7.7%	6	12.8%	12	14.5%	4	25.0%			32	11.4%
	hypoglycemia and hyperglycemia	2	1.5%			6	7.2%					8	2.9%
Total		130	100.0%	47	100.0%	83	100.0%	16	100.0%	4	100.0%	280	100.0%

**Table 11:** Serum urea—temporal evolution of values in relation to ROP stages.

ROP Stage	N	Mean	Standard Deviation	Min	Max	Median	IQR		Kruskal–Wallis Test
							25th	75th	
UREA 1									
non-ROP	130	26.82	13.164	11	75	24.00	17.00	36.00	H = 1.483
1	47	24.45	10.312	11	53	21.00	17.00	31.00	*p* = 0.830
2	83	27.19	14.033	10	92	24.00	20.00	31.00	
3	16	24.19	9.642	11	43	24.00	16.00	28.75	
A-ROP	4	26.00	15.011	13	39	26.00	13.00	39.00	
Total	280	26.37	12.812	10	92	24.00	18.00	32.00	
UREA 3									
non-ROP	130	40.62	18.283	12	100	38.00	25.75	50.00	H = 13.592
1	47	45.32	27.050	15	108	32.00	25.00	54.00	*p* = 0.009
2	83	47.19	23.911	10	120	47.00	29.00	56.00	
3	16	61.25	21.174	29	90	64.00	41.00	79.00	
A-ROP	4	43.00	12.702	32	54	43.00	32.00	54.00	
Total	280	44.57	22.236	10	120	41.50	27.00	57.00	
UREA 5									
non-ROP	130	44.29	25.295	7	130	38.00	25.00	53.75	H = 26.808
1	47	54.72	35.404	13	146	41.00	26.00	76.00	*p* < 0.001
2	83	64.43	42.216	16	186	62.00	30.00	81.00	
3	16	90.00	42.177	41	186	84.00	57.00	115.25	
A-ROP	4	41.50	6.351	36	47	41.50	36.00	47.00	
Total	280	54.59	35.707	7	186	46.00	28.25	71.00	
UREA 7									
non-ROP	128	39.23	24.637	7	139	33.00	21.00	49.00	H = 31.175
1	47	53.00	31.804	8	106	49.00	20.00	82.00	*p* < 0.001
2	83	60.89	37.498	11	192	57.00	32.00	77.00	
3	16	69.19	37.729	45	186	52.00	47.00	77.00	
A-ROP	4	44.00	5.774	39	49	44.00	39.00	49.00	
Total	278	49.82	32.440	7	192	43.00	25.00	66.00	
UREA 14									
non-ROP	112	31.57	23.351	4	137	23.00	16.00	41.00	H = 33.035
1	47	37.40	21.451	8	77	32.00	19.00	58.00	*p* < 0.001
2	83	44.14	23.815	8	156	43.00	26.00	56.00	
3	16	63.00	33.754	28	156	52.00	44.00	78.00	
A-ROP	4	29.00	2.309	27	31	29.00	27.00	31.00	
Total	262	38.48	25.017	4	156	33.00	19.00	55.00	
UREA 21									
non-ROP	101	22.70	14.758	4	86	19.00	10.00	32.50	H = 16.545
1	44	27.70	21.831	7	86	21.00	10.00	41.00	*p* = 0.002
2	83	31.64	15.962	8	79	32.00	17.00	41.00	
3	16	29.50	12.533	7	49	32.50	17.75	39.00	
A-ROP	4	25.50	6.351	20	31	25.50	20.00	31.00	
Total	248	27.06	16.765	4	86	24.00	12.00	39.00	
UREA 28									
non-ROP	77	22.29	15.541	5	68	17.00	11.00	28.00	H = 16.183
1	42	15.57	7.828	6	36	13.00	11.00	18.00	*p* = 0.003
2	83	24.67	16.316	7	72	20.00	11.00	34.00	
3	16	16.38	7.384	7	34	17.00	9.00	21.25	
A-ROP	4	39.50	14.434	27	52	39.50	27.00	52.00	
Total	222	21.79	14.768	5	72	17.00	11.00	28.00	

**Table 12:** Serum creatinine—temporal evolution of values in relation to ROP stages.

ROP Stage	N	Mean	Standard Deviation	Min	Max	Median	IQR		Kruskal–Wallis Test
							25th	75th	
CREATININE 1									
non-ROP	130	0.8744	0.45050	0.39	4.00	0.7900	0.6775	0.8900	H = 13.176
1	47	0.7349	0.19234	0.46	1.20	0.6900	0.6000	0.8500	*p* = 0.010
2	83	0.7860	0.17925	0.44	1.29	0.7500	0.6400	0.9100	
3	16	0.7181	0.12776	0.55	0.93	0.7000	0.6225	0.8500	
A-ROP	4	0.9300	0.03464	0.90	0.96	0.9300	0.9000	0.9600	
Total	280	0.8166	0.33759	0.39	4.00	0.7650	0.6525	0.8900	
CREATININE 3									
non-ROP	130	0.8326	0.47433	0.35	4.00	0.7550	0.6200	0.9725	H = 11.997
1	47	0.6981	0.25699	0.20	1.12	0.6400	0.5600	0.9700	*p* = 0.017
2	83	0.7995	0.31630	0.16	1.89	0.7800	0.5900	0.9800	
3	16	0.8456	0.27432	0.56	1.23	0.7300	0.6075	1.2200	
A-ROP	4	0.5050	0.01732	0.49	0.52	0.5050	0.4900	0.5200	
Total	280	0.7963	0.38983	0.16	4.00	0.7300	0.6000	0.9700	
CREATININE 5									
non-ROP	128	0.5748	0.26190	0.10	1.59	0.5600	0.3600	0.7300	H = 7.956
1	47	0.4804	0.23122	0.10	0.98	0.4700	0.3500	0.6400	*p* = 0.093
2	83	0.6012	0.34283	0.10	1.51	0.6100	0.3000	0.8000	
3	16	0.6788	0.30696	0.31	1.37	0.6450	0.4700	0.8525	
A-ROP	4	0.3950	0.14434	0.27	0.52	0.3950	0.2700	0.5200	
Total	278	0.5701	0.28851	0.10	1.59	0.5400	0.3500	0.7300	
CREATININE 7									
non-ROP	130	0.5145	0.24987	0.10	1.55	0.4800	0.3400	0.6100	H = 13.555
1	47	0.4274	0.25275	0.10	1.30	0.4000	0.2500	0.5000	*p* = 0.009
2	83	0.5127	0.30321	0.10	1.38	0.4600	0.3500	0.6000	
3	16	0.6025	0.24818	0.33	1.37	0.5650	0.4250	0.6975	
A-ROP	4	0.3250	0.08660	0.25	0.40	0.3250	0.2500	0.4000	
Total	280	0.5016	0.26811	0.10	1.55	0.4650	0.3325	0.6000	
CREATININE 14									
non-ROP	112	0.4762	0.15478	0.10	0.80	0.5000	0.3500	0.5975	H = 5.464
1	47	0.4715	0.16973	0.13	0.83	0.4600	0.3800	0.5800	*p* = 0.243
2	83	0.5177	0.23244	0.10	1.74	0.5000	0.3500	0.6900	
3	16	0.6325	0.41314	0.12	1.74	0.6300	0.2800	0.8100	
A-ROP	4	0.5550	0.14434	0.43	0.68	0.5550	0.4300	0.6800	
Total	262	0.4992	0.20953	0.10	1.74	0.5000	0.3600	0.6100	
CREATININE 21									
non-ROP	101	0.5245	0.24516	0.15	1.80	0.4700	0.4100	0.6150	H = 15.523
1	44	0.4666	0.17298	0.15	0.83	0.4650	0.3600	0.5900	*p* = 0.004
2	83	0.5202	0.22380	0.10	1.41	0.5200	0.4000	0.6200	
3	16	0.6988	0.19680	0.35	0.90	0.7600	0.5900	0.8600	
A-ROP	4	0.5750	0.13279	0.46	0.69	0.5750	0.4600	0.6900	
Total	248	0.5248	0.22660	0.10	1.80	0.4950	0.4000	0.6300	
CREATININE 28									
non-ROP	77	0.4827	0.13435	0.20	0.85	0.4700	0.3950	0.5900	H = 4.177
1	40	0.4588	0.14546	0.13	0.77	0.4500	0.3700	0.5300	*p* = 0.383
2	83	0.4870	0.14796	0.10	0.86	0.4800	0.3900	0.5800	
3	16	0.5713	0.18532	0.35	0.86	0.5200	0.4000	0.7650	
A-ROP	4	0.4500	0.09238	0.37	0.53	0.4500	0.3700	0.5300	
Total	220	0.4858	0.14626	0.10	0.86	0.4700	0.3900	0.5700	

## Data Availability

The original contributions presented in this study are included in the article. Further inquiries can be directed to the corresponding author(s).

## Abbreviations

The following abbreviations are used in this manuscript:

ROP	Retinopathy of prematurity
NICU	Neonatal intensive care unit
ICROP	International Classification of Retinopathy of Prematurity
ALT	Alanine aminotransferase
AST	Aspartate aminotransferase
A-ROP	Aggressive retinopathy of prematurity
GA	Gestational age
LBW	Low birth weight
ELBW	Extremely low birth weight
VEGF	Vascular Endothelial Growth Factor
IGF-1	Insulin-like Growth Factor 1
RPE	Retinal pigment epithelium
BW	Birth weight
